# Optimal Reactive Power Compensation in Distribution Networks with Radial and Meshed Structures Using D-STATCOMs: A Mixed-Integer Convex Approach

**DOI:** 10.3390/s22228676

**Published:** 2022-11-10

**Authors:** Víctor Manuel Garrido, Oscar Danilo Montoya, Ángeles Medina-Quesada, Jesus C. Hernández

**Affiliations:** 1 Programa de Ingeniería Eléctrica, Facultad de Ingenierías y Arquitectura, Universidad de Pamplona, Pamplona 543050, Colombia; 2 Grupo de Compatibilidad e Interferencia Electromagnética (GCEM), Facultad de Ingeniería, Universidad Distrital Francisco José de Caldas, Bogotá 110231, Colombia; 3 Laboratorio Inteligente de Energía, Facultad de Ingeniería, Universidad Tecnológica de Bolívar, Cartagena 131001, Colombia; 4 Department of Electrical Engineering, University of Jaén, Campus Lagunillas s/n, Edificio A3, 23071 Jaén, Spain

**Keywords:** radial and meshed distribution networks, distribution static compensators, mixed-integer convex optimization, optimal power flow approximation

## Abstract

This paper deals with the problem regarding the optimal siting and sizing of distribution static compensators (D-STATCOMs) in electrical distribution networks to minimize the expected total annual operating costs. These costs are associated with the investments made in D-STATCOMs and expected energy losses costs. To represent the electrical behavior of the distribution networks, a power flow formulation is used which includes voltages, currents, and power as variables via incidence matrix representation. This formulation generates a mixed-integer nonlinear programming (MINLP) model that accurately represents the studied problem. However, in light of the complexities involved in solving this MINLP model efficiently, this research proposes a mixed-integer convex reformulation. Numerical results regarding the final annual operating costs of the network demonstrate that the proposed mixed-integer convex model is efficient for selecting and locating D-STATCOMs in distribution networks, with the main advantage that it is applicable to radial and meshed distribution grid configurations. A comparative analysis with respect to metaheuristic optimizers and convex approximations confirms the robustness of the proposed formulation. All numerical validations were conducted in the MATLAB programming environment with our own scripts (in the case of metaheuristics) and the CVX convex disciplined tool via the Gurobi solver. In addition, the exact MINLP model is solved using the GAMS software.

## 1. Introduction

Electric distribution networks are one of the main components of electric energy systems, which are entrusted with interfacing transmission/sub-transmission systems at substations with end-users through a medium-voltage network [1]. Distribution grids typically operate between 1 and 25 kV, and they have be equivalent loads between 3 MVA and 10 MVA. These grids can also cover a few square kilometers in urban areas and hundreds of square kilometers in rural areas [2]. From a construction perspective, distribution networks are typically configured with a radial structure, i.e., there is one and only one path between each node and the substation bus [3]. Due to this electrical configuration, electric distribution grids have energy losses between 6% and 18% of the energy input at the substation terminal, which is very high when compared to high-voltage power systems, whose losses are about 2% [4].

To reduce the energy losses in distribution networks, there are different technical approaches reported in the literature, as well as those implemented by distribution companies, both with excellent results. These approaches include (i) grid reconfiguration [5], (ii) phase-balancing [6], and (iii) shunt reactive power compensation [7].

The reconfiguration strategy in distribution networks is a classical approach to minimizing grid power losses. This strategy requires the presence of tie-lines with reconfigurable devices (normally-open reclosers) in the distribution system, which work in conjunction with normally closed reclosers in order to modify the grid structure under particular load conditions [8]. Grid reconfiguration allows for excellent energy loss reduction indicators. However, it requires significant investments in protection devices and new tie-lines. In addition, the dynamic reconfiguration of distribution networks implies the use of advanced protective coordination schemes, which also increases the total grid operating costs [9].

The implementation of a phase-balancing approach for power loss reduction in distribution networks is a methodology only applicable to three-phase asymmetric networks, i.e., distribution networks with single-, two-, and three-phase loads with star or delta connections [10]. This method consists of redistributing all the load connections in all the nodes of the network, with the purpose of balancing load consumption in order to make three-phase systems operate as fully balanced systems by a close margin [11]. The phase-balancing approach is an efficient and low-cost approach for reducing energy losses in distribution networks. However, its final solution is completely dependent on load profiles, which implies that, if there are significant variations, making the phase-balancing approach efficient will require a dynamic implementation, i.e., using reconfigurable devices at each node, which will considerably increase the final costs [12].

Reactive power compensation in distribution grids can be implemented with fixed-step or variable-step capacitor banks, which constitutes a low-cost solution with important energy losses reduction effects, as well as good improvements in the grid voltage profiles [13]. However, the main problem with this solution is associated with the variability of load consumption, which implies that the injection of reactive power with fixed values is not the best option for compensating the variability of the energy losses during a typical day of operation [14]. An additional device used in distribution networks for shunt reactive power compensation is Distribution Static Compensators (D-STATCOMs), which are devices based on power electronics that have the ability to provide variable reactive power as a function of the grid requirements [15]. These devices need additional investments when compared with capacitor banks. However, their dynamic behavior regarding reactive power injection allows for better reductions in the expected annual grid energy losses, which makes these devices attractive for installation in modern distribution networks.

This research focuses on shunt reactive power injection in distribution networks using D-STATCOMs, as their dynamic behavior (dispatchable reactive power characteristics) makes these devices an excellent alternative for minimizing the annual expected energy losses in distribution networks with residential, industrial, and commercial users [16]. In the specialized literature, there are multiple approaches regarding D-STATCOMs and their optimal integration in distribution networks.

The problem regarding the optimal installation and sizing of D-STATCOMs was thoroughly studied by the authors of [17], who presented a complete review of the state of the art and outlined different methodologies for locating these devices within a distribution network. These methodologies include sensitive nodal analysis, heuristic and metaheuristic approaches, artificial neural networks, and some combinations. The authors of [18] presented the application of a discrete-continuous version of the particle swarm optimization method in order to locate and size D-STATCOMs in radial distribution networks composed of 33 and 69 nodes. Numerical results demonstrated the effectiveness of the proposed approach when compared with the vortex search algorithm, the genetic-based optimization approach, and two exact MINLP solvers available in the General Algebraic Modeling System (GAMS) software. In [19], the authors discussed the effect of the simultaneous integration of dispersed generators and D-STATCOMs in the reactive power transference capabilities of the distribution grid. The authors located and sized both devices using sensitivity-based approaches, with the main goal of maximizing the reactive loadability of the grid by keeping voltage profiles within an acceptable range of operation. The authors of [16] presented the application of the discrete-continuous version of the salp swarm algorithm to locate and size D-STATCOMs in radial and meshed distribution networks in order minimize the annual expected energy losses costs while considering the investments made in compensation devices. Numerical results in the IEEE 33-bus grid, which considered residential, industrial, and commercial loads, demonstrated the effectiveness of the proposed approach when compared to the MINLP solvers available in the GAMS software. In [20], the authors explored the problem regarding the optimal siting and sizing of D-STATCOMs in radial distribution networks while aiming to minimize the grid power losses in all the distribution branches. To select the size and location of these devices, an analytical method and the particle swarm optimization technique were employed, with promissory results in test feeders composed of 12 and 69 nodes. The authors of [21] presented the application of a mixed-integer conic reformulation to locate and size D-STATCOMs in distribution networks. The proposed formulation was tested in the IEEE 33-bus grid, but it is only applicable to purely radial distribution networks, since it is based on the branch power flow approach presented in [22]. In [23], the authors explored the aforementioned problem by using a probabilistic approach and sensitive factors. The main goal of this research was improving the voltage stability margin throughout the distribution network. Monte Carlo simulations demonstrated the effectiveness of the proposed approach in the IEEE 85-bus system. In [24], an analytical approach to locate and size D-STATCOMs and dispersed sources was presented. The authors used the stability index and the loss-sensitive factor in order to determine the best locations and sizes for the dispersed generators and D-STATCOMs. Numerical results in the IEEE 33-bus grid demonstrated the effectiveness of the proposed approach with regard to the comparison between the benchmark case and the final power losses and voltage profiles. Other optimization methods available in the current literature to locate and size D-STATCOMs in distribution networks include the fuzzy-lightning search algorithm [25], the differential evolution algorithm [26], the whale optimization algorithm [27], the bat optimization algorithm [28], the tabu search algorithm, the ant colony optimizer [29], and the ant lion optimizer [30], among others.

Table 1 summarizes the main approaches reported in the current literature to address the problem of optimally placing and sizing D-STATCOMs in distribution networks.

The main characteristics of the summarized optimization approaches for locating and sizing D-STATCOMs in distribution networks are the following: (i) most of the literature reports focus on the power losses minimization and voltage profile improvements; (ii) three tendencies to solve the optimization problem can be identified, i.e., selection of nodes and sizes using sensitive index, the application of combinatorial optimization methods, and the application of convex-based approaches; and (iii) the problem regarding location and sizes of D-STATCOMs continues being a studied optimization problem with recent publications in the last decade. Based on the above, this research makes the following contributions:

- The proposal of a new two-stage approach to the problem under study by using a mixed-integer convex model to select the optimal nodes for locating the D-STATCOMs and a nonlinear programming model to determine their optimal sizes.
- The applicability of the proposed modeling to radial and meshed distribution grids without any modification in their mathematical structure, with the main advantage that different users are considered, i.e., residential, industrial, and commercial loads.

It is worth mentioning that this research focuses on using D-STATCOMs in radial and meshed distribution networks at medium-voltage levels while aiming to minimize the annual expected energy losses costs and considering D-STATCOM investments in the objective function. However, the maintenance and operating costs of the D-STATCOMs are neglected in this research, as these devices require very few revisions/interventions during their useful life, which can be between 15 and 25 years. In addition, this research only considers the typical daily load behavior of a work day that includes residential, industrial, and commercial users connected to the distribution grid. This was based on the fact that the distribution grid is located in a tropical country, i.e., between the tropics of Capricorn and Cancer, as is the case of Colombia [16]. Notwithstanding, more research is needed to include the seasonal behavior of the demand in the expected placement and sizing of D-STATCOMs.

The remainder of this document is structured as follows: Section 2 presents the exact mathematical modeling of the studied problem in its general mixed-integer nonlinear programming (MINLP) form, Section 3 presents the main considerations for converting the MINLP formulation into a mixed-integer convex equivalent, in order to determine the nodes where the D-STATCOMs will be located; Section 4 describes the solution methodology regarding the selection of the nodes and the optimal sizes of the D-STATCOMs by using a nonlinear programming evaluator; Section 5 shows the main characteristics of the distribution system considered in this study, i.e., the IEEE 33-bus grid, along with its radial and meshed configurations and its load distribution per area; Section 6 presents all the numerical validations of the proposed solution methodology, as well as its comparison with metaheuristic optimization methods; and Section 7 lists the main conclusions derived from this research, as well as some possible future works.

## 2. Exact MINLP Formulation

The problem regarding the optimal placement and sizing of D-STATCOMs in electric distribution networks involves a MINLP model that combines binary variables regarding the nodes where the D-STATCOMs will be located and continuous variables regarding voltages, currents, power flows, and D-STATCOM sizes, among others. The exact MINLP model that represents the studied problem is presented in Equations (1)–(9).

(1) $$\begin{array}{cc} \mathrm{Obj}.\;\mathrm{fun}.: & \;\\ \; & \;\min z=\sigma T\sum_{l\in L}\sum_{h\in H}R_{l}\left({\left(i_{l,h}^{r}\right)}^{2}+{\left(i_{l,h}^{i}\right)}^{2}\right)\Delta h+\delta T\sum_{j\in N}m_{j}\left(\alpha m_{j}^{2}+\beta m_{j}+\gamma \right) \end{array}$$

where *z* represents the objective function value regarding the expected annual operating costs of the distribution grid, σ corresponds to the average energy cost for the distribution system’s operator, *T* represents the number of days in an ordinary year, $R_{l}$ means the resistive effect associated with the distribution line *l*, $i_{l,h}^{r}$ and $i_{l,h}^{i}$ are the real and imaginary components of the current flowing through the line *l* in the period of time *h*, Δh represents the period of time where electrical variables are assumed to be constant, δ is the cost factor regarding the installation of the D-STATCOMs, $m_{j}$ means the size of a D-STATCOM installed at node *j*, and α, β, and γ correspond to the cubic, quadratic, and linear cost coefficients associated with the investments made in D-STATCOMs, respectively.

**Remark** **1.** *The selection of the cubic function that represents the expected investment costs of the D-STATCOMs presented in the second part of Equation (1) was made based on the recommendations made by the authors of [31,37], who presented a general formulation for integrating D-STATCOMs while considering their annualized investment costs and a planning period of 5 years.*

(2) $$\begin{array}{cc} \mathrm{Subject}\;\mathrm{to}.: & \;\\ \; & \;p_{j,h}^{g}-P_{j,h}^{d}=\sum_{l\in L}A_{jl}\left(V_{j,h}^{r}i_{l,h}^{r}+V_{j,k}^{i}i_{l,h}^{i}\right),\;\left\{\forall j\in N,\;\forall h\in H\right\}, \end{array}$$

(3) $$\begin{array}{cc} \; & \;q_{j,h}^{g}+q_{j,h}^{st}-Q_{j,h}^{d}=-\sum_{l\in L}A_{jl}\left(V_{j,h}^{r}i_{l,h}^{i}-V_{j,h}^{i}i_{l,h}^{r}\right),\;\left\{\forall j\in N,\;\forall h\in H\right\}, \end{array}$$

(4) $$\begin{array}{cc} \; & \;0\leq m_{j}\leq y_{j}q^{\max},\;\left\{\forall j\in N\right\}, \end{array}$$

(5) $$\begin{array}{cc} \; & \;-m_{j}\leq q_{j,h}^{st}\leq m_{j},\;\left\{\forall j\in N,\forall h\in H\right\}, \end{array}$$

(6) $$\begin{array}{cc} \; & \;\sum_{j\in N}y_{j}\leq \eta , \end{array}$$

(7) $$\begin{array}{cc} \; & \;y_{k}\in \left\{0,1\right\}\;\left\{\forall k\in N\right\}, \end{array}$$

(8) $$\begin{array}{cc} \; & \;\begin{array}{c} V_{\min}\leq \sqrt{{\left(V_{j,h}^{r}\right)}^{2}+{\left(V_{j,h}^{i}\right)}^{2}}\leq V_{\max},\;\left\{\forall j\in N,\forall h\in H\right\}, \end{array} \end{array}$$

(9) $$\begin{array}{cc} \; & \;\begin{array}{c} 0\leq \sqrt{{\left(i_{l,h}^{r}\right)}^{2}+{\left(i_{l,h}^{i}\right)}^{2}}\leq I_{l,\max},\;\left\{\forall l\in L,\forall h\in H\right\}, \end{array} \end{array}$$

where $p_{j,h}^{g}$ corresponds to the active power generation in the slack source connected at node *j* in the period of time *h*, $P_{j,h}^{d}$ represents the demanded active power at node *j* in the period of time *h*, $V_{j,h}^{r}$ and $V_{j,h}^{i}$ are the real and imaginary parts of the voltage profiles at node *j* for each period of time, $A_{jl}$ corresponds to the component of the node-to-branch matrix that relates the node *j* with the line *l*, $q_{j,h}^{g}$ means the reactive power injection in the slack source at node *j* in the period of time *h*, $q_{j,h}^{st}$ represents the reactive power injection provided by the D-STATCOM connected at node *j* in the period of time *h*, $Q_{j,h}^{d}$ represents the demanded reactive power at node *j* in the period of time *h*, $y_{j}$ represents a binary decision variable regarding the placement ($y_{j}=1$) or not ($y_{j}=0$) of a D-STATCOM at node *j*, $q^{\max}$ means the maximum size allowed for shunt reactive power compensation, η is the maximum number of D-STATCOMs available for integration in the distribution grid. $V_{\min}$ and $V_{\max}$ represent the minimum and maximum voltage regulation bounds allowed for all the voltage profiles in the distribution network in any period of time, and $i_{l,\max}$ corresponds to the maximum thermal current allowed in the conductor associated with route *l* in any period of time.

Note that L corresponds to the set that contains all the distribution lines of the network.

For the set of constraints (2)–(9) it is important to highlight that:

- Equations (2) and (3) are widely known in electrical engineering as power balance constraints, which make sure that the power input injections and absorptions at a particular node are equal to the flow sent/received to/from the distribution lines, i.e., it is the application of the Tellegen theorem regarding power equilibrium in electrical networks.
- Inequality constraint (4) reveals the positive nature of the $m_{j}$ variable, which has to do with the nominal power rate of the D-STATCOM device assigned at node *j*. However, the box-type constraint (5) shows that the D-STATCOM assigned at node *j* has the ability to inject/absorb reactive power to/from the distribution grid, i.e., it is a flexible device that works with variable power factors, including lagging or leading, as a function of the grid requirements.
- Inequality constraints (6) and (7) refer to the number of D-STATCOMS available to be installed along with the distribution network, and they confirm the binary nature of the decision variable associated with the location of the D-STATCOMs in the distribution grid.
- Inequality constraints (8) and (9) ensure that voltage regulation is performed at all the nodes of the network in all periods of time, and that the current flowing through the corridor *l* is contained within the thermal limits of its conductor.

**Remark** **2.** *The main characteristics of the optimization model (1)–(9) are the following: (i) the objective function is nonlinear and non-convex due to the cubic form of the costs of the D-STATCOMs, (ii) the power balance constraints are also nonlinear due to the product among voltages and currents on the right-hand-side part of Equations (2) and (3), and (iii) the voltage regulation constraint (8) and the thermal current limits defined in (9) are nonlinear due to the presence of root-square functions.*

Note that, to deal with the MINLP structure of the studied problem, the current literature presents two alternatives. The first approach corresponds to the application of combinatorial optimization methods using a master-slave solution methodology, where the master-stage defines the location and size of the D-STATCOMs and the slave stage solves the power flow problem [16]. The second approach is based on approximating the MINLP model using mixed-integer convex theory in order to reach the optimal solution of the problem via the Branch and Cut technique, which is combined with interior point methods [21]. This research proposes a mixed-integer convex equivalent optimization model to locate and size D-STATCOMs in radial and meshed distribution networks while considering different demand behaviors, i.e., residential, commercial, and industrial loads. The proposed convex equivalent reformulation will be presented in next section.

## 3. Mixed-Integer Convex Reformulation

The optimization model that represents the problem regarding the optimal location and sizing of D-STATCOMs in distribution networks defined in Equations (1)–(11) is, as previously discussed, an MINLP problem, which makes its solution with conventional optimization methods a difficult matter, due to the high probability of getting stuck in local optima [38].

To deal with the complexities of the MINLP modeling, this study proposes a simple mixed-integer quadratic convex (MIQC) approximation based on the following facts:

- The quadratic component of the objective function regarding the expected costs of the energy losses is indeed a convex function due to the fact that the resistive parameter of the distribution line is a positive definite parameter.
- The component of the objective function regarding the investment costs in D-STATCOMs is a non-convex function owing to the presence of a cubic function combined with the signs of the parameters α, β, and γ. However, as discussed by the authors of [21], because the value of $m_{j}$ is lower than 2 Mvar for distribution networks, and given the small values of the parameters α and β, more than 95% of the cost of the D-STATCOMs is only dependent on its linear component. Therefore, the investment costs related to D-STATCOMs can be transformed into a linear (convex) component.
- In the set of constraints, only the power balance Equations (2) and (3) are nonlinear and non-convex. However, these can be approximated as linear convex constraints by relaxing the voltage values, i.e., by previously solving a power flow problem without considering D-STATCOMs. The solution to the power flow problem will provide an approximation of the voltage profiles in all nodes of the network for all the period of times under study, which will have soft variations once the D-STATCOMs are installed.
- The voltage regulation constraint (8) is relaxed in the MIC formulation, as the voltage magnitudes are assumed to be known. The current limit constraint (9) is reformulated as a conic constraint using the $l_{2}$-norm, i.e., a convex constraint [22].

Considering the aforementioned assumptions, the proposed MIQC model to determine the optimal location and size of D-STATCOMs in radial and meshed distribution networks takes the following form:

(10) $$\begin{array}{cc} \mathrm{Objective}\;\mathrm{function}: & \;\\ \; & \;\min z_{\mathrm{approx}}=\sigma T\sum_{l\in L}\sum_{h\in H}R_{l}\left({\left(i_{l,h}^{r}\right)}^{2}+{\left(i_{l,h}^{i}\right)}^{2}\right)\Delta h+\delta T\sum_{j\in N}\gamma m_{j} \end{array}$$

(11) $$\begin{array}{cc} \mathrm{Subject}\;\mathrm{to}.: & \;\\ \; & \;p_{j,h}^{g}-P_{j,h}^{d}=\sum_{l\in L}A_{jl}\left(V_{j,h}^{r,0}i_{l,h}^{r}+V_{j,k}^{i,0}i_{l,h}^{i}\right),\;\left\{\forall j\in N,\;\forall h\in H\right\}, \end{array}$$

(12) $$\begin{array}{cc} \; & \;q_{j,h}^{g}+q_{j,h}^{st}-Q_{j,h}^{d}=-\sum_{l\in L}A_{jl}\left(V_{j,h}^{r,0}i_{l,h}^{i}-V_{j,h}^{i,0}i_{l,h}^{r}\right),\;\left\{\forall j\in N,\;\forall h\in H\right\}, \end{array}$$

(13) $$\begin{array}{cc} \; & \;0\leq m_{j}\leq y_{j}q^{\max},\;\left\{\forall j\in N\right\}, \end{array}$$

(14) $$\begin{array}{cc} \; & \;-m_{j}\leq q_{j,h}^{st}\leq m_{j},\;\left\{\forall j\in N\right\},\forall h\in H, \end{array}$$

(15) $$\begin{array}{cc} \; & \;\sum_{j\in N}y_{j}\leq \eta , \end{array}$$

(16) $$\begin{array}{cc} \; & \;y_{k}\in \left\{0,1\right\}\;\left\{\forall k\in N\right\}, \end{array}$$

(17) $$\begin{array}{cc} \; & \;\begin{array}{c} ∥i_{l,h}^{r},\;i_{l,h}^{i}∥\leq I_{l,\max}.\;\left\{\forall l\in L,\forall h\in H\right\}, \end{array} \end{array}$$

where $V_{j,h}^{r,0}$ and $V_{j,h}^{i,0}$ represent the initial values assigned to the real and imaginary parts of the voltage profiles after solving the multi-period power flow problem, respectively.

**Remark** **3.** *To ensure that the solution provided by the MIQC model is 100% feasible in the exact MINLP model, the solutions of the $y_{j}$ variables (locations of the D-STATCOMs) provided by the MIQC model are fixed in the MINLP one in order to refine their optimal sizes and calculate the exact value of the objective function.*

## 4. Summary of the Optimization Methodology

**Figure 1:**
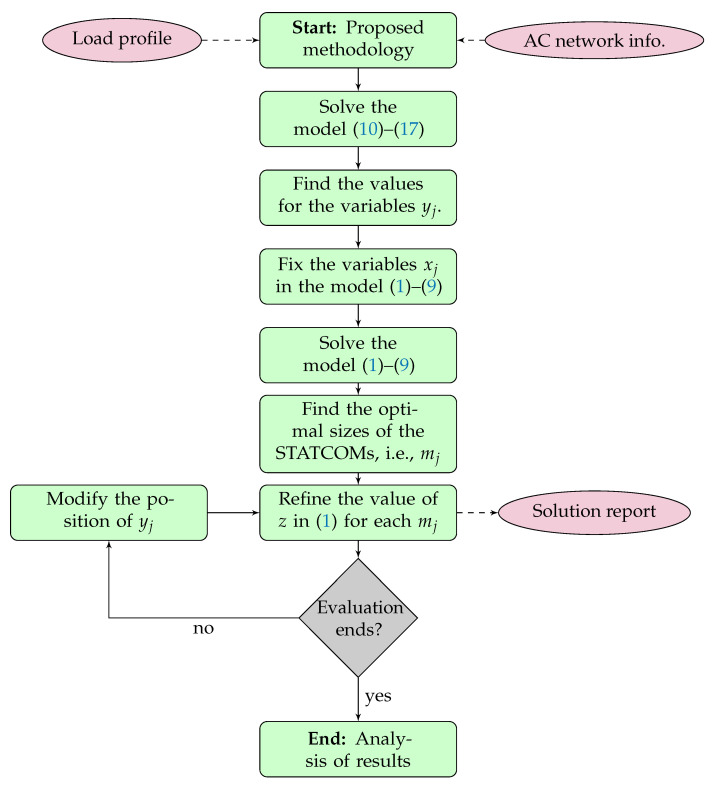
Main aspects of the proposed solution methodology.

To solve the problem regarding the optimal siting and sizing of D-STATCOMs in radial and meshed distribution networks, a two-stage approach is implemented. In the first stage, the proposed MIQC model is solved in order to determine the nodes ($y_{j}$) where the D-STATCOMs must be located. In the second stage, these binary variables are set in the MINLP model (1)–(9) which then becomes a nonlinear programming model (NLP). This NLP model is solved using a specialized interior point method to refine the values of the D-STATCOMs sizes ($m_{j}$) and find the exact objective function value. The summary of the proposed solution methodology is presented in Figure 1.

**Remark** **4.** *It is worth mentioning that the proposed optimization methodology based on the two-stage approach summarized in Figure 1 is totally independent of the optimization tool, with the main advantage that it can be implemented in any optimization software that can solve MIQC models [39].*

## 5. Test Feeder Information

**Figure 2:**
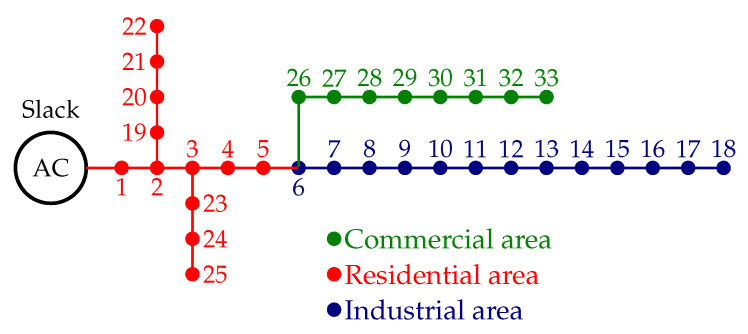
Schematic configuration of the IEEE 33-bus grid with load classifications.

To validate the effectiveness and robustness of the proposed two-stage optimization approach in siting and sizing D-STATCOMs in radial and meshed distribution networks, the IEEE 33-bus grid is employed as a test feeder [16]. The schematic configuration of this grid is depicted in Figure 2.

This test feeder is operated at the substation bus with a nominal voltage of 12.66 kV. Its parametric information regarding peak load consumptions and distribution lines is listed in Table 2.

With the purpose of evaluating the effect of load areas on the optimal location and sizing of the D-STATCOMs in the distribution grid, the daily behavior of residential, industrial and commercial loads is listed in Table 3.

To evaluate the objective function defined in Equation (1), the parameters reported in Table 4 are considered, which were taken from [16]. To avoid mistakes during the evaluation of this objective function, it is important to mention that the variable $m_{j}$ must be defined in Mvar.

## 6. Numerical Validations

The implementation of the proposed mixed-integer optimization model to define the nodes where the D-STATCOMs must be placed was conducted in the MATLAB programming environment with the CVX tool and the Gurobi solver. In order to refine the size of these D-STATCOMs, the GAMS software with the BONMIN solver was employed. Note that all the implementations were carried out on a PC with an AMD Ryzen 7 3700 2.3-GHz processor and 16.0 GB RAM, running a 64-bit version of Microsoft Windows 10 Single Language.

Our methodology was compared to the most recent results in this research area, which were obtained by [16] through the application of a solution methodology based on the salp swarm algorithm (SSA). For this comparison, two simulation cases were considered: (i) the injection of fixed-reactive power, i.e., operation as fixed-step capacitor banks; and (ii) variable reactive power injection. Note that the GAMS software was also used to solve the exact MINLP model presented in Equations (1)–(9).

### 6.1. Solution for the IEEE 33-Bus Grid with a Radial Configuration

Table 5 presents all numerical comparisons for the IEEE 33-bus grid when the reactive power injection in the D-STATCOMs is considered to be constant throughout the operation of the distribution network.

The numerical results listed in Table 5 show that:

- The proposed optimization model based on the combination of the MIC and the NLP approaches allows finding a better optimal solution in comparison with the SSA approach. The proposed approach found an additional gain of USD 33.42 (i.e., 0.03%) regarding the SSA approach. Note that, with respect to the benchmark case, our proposal found an expected annual gain of USD 22,397.96. This result clearly demonstrates that the use of D-STATCOMs in distribution networks could be considered an efficient alternative to reduce the expected costs of energy losses, even if these devices are operated as fixed-step capacitor banks.
- The solution of the exact MINLP model with the GAMS-based BONMIN and COUENNE solvers clearly demonstrates that, due to the non-convex nature of the original optimization model (see Equations (1)–(9)), both solvers got stuck in local optima. This evinces the needed for using efficient solution methods to deal with the problem of optimal reactive power compensation in distribution networks.
- Regarding the final nodes selected for the optimal siting of D-STATCOMs, the SSA and BONMIN approaches, as well as the proposed methodology, identified node 30 as the most promising node to place a D-STATCOM larger than those in the remaining nodes. In addition, when comparing the SSA approach with the proposed MIC-NLP method, the main difference between both solutions is the variation in the location of a D-STATCOM (namely from node 13 to node 14). Note that the total reactive power installation required by the SSA approach was 891.99 kvar, while the proposed approach installed about 869.83 kvar, which implies that, by injecting less reactive power better selected in nodes, it is possible to reach better objective function values.

To illustrate the main advantage of using D-STATCOMs in distribution networks, i.e., the possibility of injecting variable reactive power in all daily operation time steps as a function of the demand behavior, Table 6 presents the solution for all the locations provided in Table 5. Note that, in this simulation case, the nodes for the D-STATCOMs reported in the second column of Table 5 are fixed as inputs for the NLP model (1)–(9).

The results in Table 6 show that:

- For all solution methodologies, when the reactive power injection is variable for a daily operation scenario, improvements were found with respect to the fixed operation cases (Table 5). Note that the SSA approach found additional reductions of USD 1669.67, and the proposed MIC-NLP approach found additional reductions of USD 1665.25. This means that both approaches evidence that variable reactive power injection in the distribution grid allows for additional improvements regarding the final objective function value in comparison with the fixed-operation case.
- D-STATCOM sizing increases when variable reactive power injection is considered. The nominal size of all the D-STATCOMs in the fixed-injection scenario was 891.99 kvar, which increased to 1038.55 kvar for the variable operation case. For the proposed approach, when the fixed case is considered, the total size of the D-STATCOMs was 869.83 kvar, which increased to 1034.67 kvar. These results are expected since, for a dynamic behavior, an additional reserve of power injection is required for periods of time with maximum demand behavior. Moreover, note that the solution provided by the proposed MIC-NLP approach uses less installed capabilities when compared to the SSA approach. However, a better objective function value was found. This demonstrates that the selection of the set of nodes for locating D-STATCOMs is essential to finding the optimal solution, which mainly depends on the strong nonlinear relation between the decision variables (both binary and continuous ones).

### 6.2. Solution for the IEEE 33-Bus Grid with a Meshed Configuration

**Figure 3:**
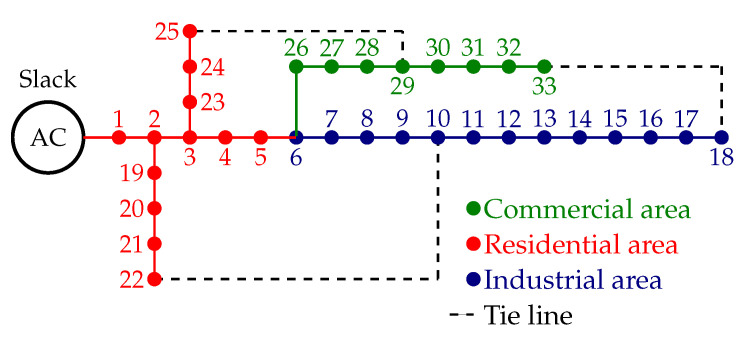
Schematic meshed configuration of the IEEE 33-bus grid with load classifications.

To evaluate the numerical performance of the proposed approach in the meshed configuration, the addition of three lines to the radial configuration presented in Figure 2 was considered. These lines are between nodes 10 and 22, 18 and 33, and 25 and 29, with resistances and reactances of $\left(2+j2\right)\;\Omega$, $\left(0.50+j0.50\right)\;\Omega$, and $\left(0.50+j0.50\right)\;\Omega$, respectively (the schematic meshed configuration for the IEEE 33-bus grid with meshed structure is presented in Figure 3).

Table 7 presents the numerical results for the SSA approach, the GAMS solvers, and the proposed MIC-NLP approach considering fixed reactive power injection.

The results in Table 7 show that:

- The proposed MIC-NLP approach reaches the best objective function value, with a reduction of about 10.45% with respect to the benchmark case, by locating D-STATCOMs at nodes 10, 30, and 32, with a total size of 731.82 kvar. Note that this solution is only followed by the results reached with the SSA approach, which achieved a reduction of 10.41% by locating the D-STATCOMs at the same nodes as our proposal, with a total size of 742.90 kvar. The difference between both solutions is given by the combinatorial nature of the SSA approach, with which it is not possible to ensure that the global optimum is found, unlike the proposed MIC-NLP methodology.
- Owing to the non-convex nature of the exact MINLP model (as in the radial case), the GAMS solvers COUENNE and BONMIN got stuck in local optima, with reductions of 4.21% and 6.61% regarding the benchmark case, respectively. The proposed approach improved these solutions by about 6.24% and 3.84%, respectively, which demonstrates the need for presenting efficient approaches to locate and size D-STATCOMs in distribution networks.
- As expected for meshed configurations, the reductions regarding annual energy losses costs were considerably lower when compared to the radial case for all the analyzed solution methods. This is explained by the meshed configuration of the network, which allows for better flow redistribution along the distribution lines, thus reducing energy losses and improving voltage profiles [40].

To confirm the applicability of using the proposed approach for dispatching variable reactive using D-STATCOMs in meshed grid configurations, the nodal locations provided in Table 7 were fixed in the MINLP model and evaluated as listed in Table 8.

The results in Table 8 show that:

- As expected, with variable reactive power injection, the MIC-NLP and the SSA approach found the same optimal solution, as the set of nodes selected for the D-STATCOMs was the same. In this scenario, the net savings obtained by implementing the D-STATCOMs were USD 9217.21 with respect to the benchmark case.
- The solution reached by the COUENNE solver in both simulation scenarios was the same in terms of the objective function value (see the second row in Table 7 and Table 8), which demonstrates that this solver remains effectively trapped in local optima due to the complexity of the solution space for the MINLP formulation.

## 7. Conclusions and Future Work

The problem regarding the optimal siting and sizing of D-STATCOMs in radial and meshed distribution networks was addressed in this research by applying a two-stage optimization approach. In the first stage, the exact MINLP model was reduced to a mixed-integer convex approximation, which allowed determining the best set of nodes for locating the D-STATCOMs. In the second optimization stage, the optimal sizes of these devices were found by solving the nonlinear programming model obtained by fixing all the binary variables. Numerical results in the IEEE 33-bus grid showed that:

- For the radial configuration, the proposed model found the best solution, reducing the expected energy losses costs by about 17.15% for the fixed power injection scenario and 18.42% for the variable reactive power injection case. These reductions were possible when the D-STATCOMs were located at nodes 14, 25, and 30. The second best approach was the SSA method, as recently reported in the scientific literature, with equivalent reductions of about 17.12 and 18.40%, respectively.
- As for the meshed configuration, the proposed optimization approach reduced the expected energy losses costs by about 10.45% for the fixed reactive power injection scenario and 10.60% for the variable reactive power injection case. These reductions confirmed the effectiveness of the proposed two-stage approach in siting and sizing D-STATCOMs in radial and meshed distribution networks when compared to the SSA-based solution methodology.
- Numerical simulations considering the variable reactive power injection capabilities of D-STATCOMs confirmed that compensating reactive power using variable injection allows for better reductions in the final expected costs of energy losses. This is explained the by the possibility of varying the reactive power injection as a function of grid requirements, i.e., as a function of residential, industrial, and commercial load profiles.

As future work, it will be possible to make the following contributions: (i) extending the proposed MIC-NLP formulation to the problem of optimally siting and sizing renewable energy resources in distribution grids; (ii) combining D-STATCOMs with renewable energy resources in order to improve the investment and operating costs for distribution companies over a planning period between 5 and 20 years; (iii) extending the proposed MIC-NLP approach to locate and size batteries in distribution networks; and (iv) considering the seasonal behavior of residential, industrial, and commercial users, as well as the variability of renewable generation sources for electrical systems located in countries with significant weather- or season-related variations in electrical consumption/generation.

## Figures and Tables

**Table 1:** Recent literature reports associated with the problem regarding the optimal siting and sizing of D-STATCOMs in distribution networks.

Solution Methodology	Objective Function	Ref.	Year
Particle swarm optimization	Power losses minimization, voltage profile improvement, and line loadability improvement	[20,31,32]	2005, 2014, 2018
Differential evolution algorithm	Power losses minimization and voltage profile improvement	[33]	2011
Ant Colony and Ant Lion optimizers	Power losses minimization and voltage profile improvement	[29,30]	2013, 2019
Sensitive index based on reactive power	Power losses minimization and voltage stability improvement	[23,34,35]	2015, 2018, 2019
Evolution-based Bat algorithm	Power losses minimization and voltage profile improvement	[28]	2021
Mixed convex-genetic approach	Energy losses and investments costs minimization	[21,36]	2021, 2022
Salp Swarm algorithm	Energy losses and investment costs minimization	[16]	2022

**Table 2:** Parametric information of the IEEE 33-bus grid regarding loads and distribution lines.

Node *i*	Node *j*	$R_{\mathrm{ij}}$ (Ω)	$X_{\mathrm{ij}}$ (Ω)	$P_{j}$ (kW)	$Q_{j}$ (kvar)	Node *i*	Node *j*	$R_{\mathrm{ij}}$ (Ω)	$X_{\mathrm{ij}}$ (Ω)	$P_{j}$ (kW)	$Q_{j}$ (kvar)
1	2	0.0922	0.0477	100	60	17	18	0.7320	0.5740	90	40
2	3	0.4930	0.2511	90	40	2	19	0.1640	0.1565	90	40
3	4	0.3660	0.1864	120	80	19	20	1.5042	1.3554	90	40
4	5	0.3811	0.1941	60	30	20	21	0.4095	0.4784	90	40
5	6	0.8190	0.7070	60	20	21	22	0.7089	0.9373	90	40
6	7	0.1872	0.6188	200	100	3	23	0.4512	0.3083	90	50
7	8	1.7114	1.2351	200	100	23	24	0.8980	0.7091	420	200
8	9	1.0300	0.7400	60	20	24	25	0.8960	0.7011	420	200
9	10	1.0400	0.7400	60	20	6	26	0.2030	0.1034	60	25
10	11	0.1966	0.0650	45	30	26	27	0.2842	0.1447	60	25
11	12	0.3744	0.1238	60	35	27	28	1.0590	0.9337	60	20
12	13	1.4680	1.1550	60	35	28	29	0.8042	0.7006	120	70
13	14	0.5416	0.7129	120	80	29	30	0.5075	0.2585	200	600
14	15	0.5910	0.5260	60	10	30	31	0.9744	0.9630	150	70
15	16	0.7463	0.5450	60	20	31	32	0.3105	0.3619	210	100
16	17	1.2890	1.7210	60	20	32	33	0.3410	0.5302	60	40

**Table 3:** Daily demand profiles for all the areas depicted in Figure 2.

Hour	Ind. (pu)	Res. (pu)	Com. (pu)
1	0.56	0.69	0.20
2	0.54	0.65	0.19
3	0.52	0.62	0.18
4	0.50	0.56	0.18
5	0.55	0.58	0.20
6	0.58	0.61	0.22
7	0.68	0.64	0.25
8	0.80	0.76	0.40
9	0.90	0.90	0.65
10	0.98	0.95	0.86
11	1	0.98	0.90
12	0.94	1	0.92
13	0.95	0.99	0.89
14	0.96	0.99	0.92
15	0.9	1	0.94
16	0.83	0.96	0.96
17	0.78	0.96	1
18	0.72	0.94	0.88
19	0.71	0.93	0.76
20	0.70	0.92	0.73
21	0.69	0.91	0.65
22	0.67	0.88	0.5
23	0.65	0.84	0.28
24	0.60	0.72	0.22

**Table 4:** Parameters for evaluating the installation costs of the D-STATCOMs.

Par.	Value	Unit	Par.	Value	Unit
$C_{\mathrm{kWh}}$	0.1390	US$/kWh	*T*	365	Days
$\Delta_{h}$	1.00	h	α	0.30	US$/MVAr${}^{3}$
β	−305.10	US$/MVAr${}^{2}$	γ	127,380	US$/MVAr
δ	6/21,900	1/Days-years	-	-	-

**Table 5:** Optimal solutions reached by the comparison and proposed optimization methods with fixed reactive power injection in a radial grid configuration.

Method	Nodes	Sizes (Mvar)	Annual Costs (USD/Year)	Red. (%)
Ben. Case	-	-	130,613.90	0.00
COUENNE	$\left[\begin{array}{ccc} 5 & 6 & 11 \end{array}\right]$	$\left[\begin{array}{ccc} 0.0000 & 0.53600 & 0.27466 \end{array}\right]$	115,960,99	11.22
BONMIN	$\left[\begin{array}{ccc} 8 & 25 & 30 \end{array}\right]$	$\left[\begin{array}{ccc} 0.29796 & 0.09204 & 0.51265 \end{array}\right]$	109,560.85	16.12
SSA	$\left[\begin{array}{ccc} 13 & 25 & 30 \end{array}\right]$	$\left[\begin{array}{ccc} 0.25851 & 0.10547 & 0.52801 \end{array}\right]$	108,249.36	17.12
MIC-NLP	$\left[\begin{array}{ccc} 14 & 25 & 30 \end{array}\right]$	$\left[\begin{array}{ccc} 0.23082 & 0.09996 & 0.53905 \end{array}\right]$	108,215.94	17.15

**Table 6:** Optimal solutions reached by the comparison and proposed optimization methods with variable reactive power injection in a radial grid configuration.

Method	Nodes	Sizes (Mvar)	Annual Costs (USD/Year)	Red. (%)
Ben. Case	-	-	130,613.90	0.00
COUENNE	$\left[\begin{array}{ccc} 5 & 6 & 11 \end{array}\right]$	$\left[\begin{array}{ccc} 0.0000 & 0.58657 & 0.30960 \end{array}\right]$	115,606.43	11.49
BONMIN	$\left[\begin{array}{ccc} 8 & 25 & 30 \end{array}\right]$	$\left[\begin{array}{ccc} 0.30077 & 0.09308 & 0.66973 \end{array}\right]$	107,902.99	17.39
SSA	$\left[\begin{array}{ccc} 13 & 25 & 30 \end{array}\right]$	$\left[\begin{array}{ccc} 0.24895 & 0.10020 & 0.68940 \end{array}\right]$	106,579.39	18.40
MIC-NLP	$\left[\begin{array}{ccc} 14 & 25 & 30 \end{array}\right]$	$\left[\begin{array}{ccc} 0.24092 & 0.10118 & 0.69257 \end{array}\right]$	106,550.69	18.42

**Table 7:** Optimal solutions reached by the comparison and proposed optimization methods with fixed reactive power injection in a meshed grid configuration.

Method	Nodes	Sizes (Mvar)	Annual Costs (USD/Year)	Red. (%)
Ben. Case	-	-	86,914.74	0.00
COUENNE	$\left[\begin{array}{ccc} 5 & 6 & 11 \end{array}\right]$	$\left[\begin{array}{ccc} 0.0000 & 0.32577 & 0.23872 \end{array}\right]$	83,254.95	4.21
BONMIN	$\left[\begin{array}{ccc} 15 & 16 & 17 \end{array}\right]$	$\left[\begin{array}{ccc} 0.11818 & 0.00391 & 0.34700 \end{array}\right]$	81,171.76	6.61
SSA	$\left[\begin{array}{ccc} 14 & 30 & 32 \end{array}\right]$	$\left[\begin{array}{ccc} 0.14620 & 0.39440 & 0.20230 \end{array}\right]$	77,870.17	10.41
MIC-NLP	$\left[\begin{array}{ccc} 14 & 30 & 32 \end{array}\right]$	$\left[\begin{array}{ccc} 0.11554 & 0.46481 & 0.15147 \end{array}\right]$	77,834.42	10.45

**Table 8:** Optimal solutions reached by the comparison and proposed optimization methods with variable reactive power injection in a meshed grid configuration.

Method	Nodes	Sizes (Mvar)	Annual Costs (USD/Year)	Red. (%)
Ben. Case	-	-	86,914.74	0.00
COUENNE	$\left[\begin{array}{ccc} 5 & 6 & 11 \end{array}\right]$	$\left[\begin{array}{ccc} 0.00000 & 0.32578 & 0.23872 \end{array}\right]$	83,254.95	4.21
BONMIN	$\left[\begin{array}{ccc} 15 & 16 & 17 \end{array}\right]$	$\left[\begin{array}{ccc} 0.11835 & 0.00396 & 0.37393 \end{array}\right]$	81,118.73	6.67
SSA	$\left[\begin{array}{ccc} 14 & 30 & 32 \end{array}\right]$	$\left[\begin{array}{ccc} 0.11578 & 0.49915 & 0.17417 \end{array}\right]$	77,697.53	10.60
MIC-NLP	$\left[\begin{array}{ccc} 14 & 30 & 32 \end{array}\right]$	$\left[\begin{array}{ccc} 0.11578 & 0.49915 & 0.17417 \end{array}\right]$	77,697.53	10.60

## Data Availability

No new data were created or analyzed in this study. Data sharing is not applicable to this article.
